# Seasonal but not sex-biased gene expression of the carotenoid ketolase, *CYP2J19*, in the sexually dichromatic southern red bishop (*Euplectes orix*)

**DOI:** 10.1098/rsos.220434

**Published:** 2022-08-03

**Authors:** Willow R. Lindsay, Rute Mendonça, Mathilda Waleij Slight, Maria Prager, Mats X. Andersson, Nicholas I. Mundy, Staffan Andersson

**Affiliations:** ^1^ Department of Biological and Environmental Sciences, University of Gothenburg, Medicinaregatan 18, SE-413 -90 Gothenburg, Sweden; ^2^ Department of Ecology, Environment and Plant Sciences, University of Stockholm, 10691 Stockholm, Sweden; ^3^ Department of Zoology, University of Cambridge, Cambridge CB2 3EJ, UK

**Keywords:** carotenoid metabolism, ketocarotenoids, plumage coloration, *CYP2J19*, testosterone, androstenedione

## Abstract

Intense red colours in birds are often owing to ketocarotenoids (KCs). In many land birds, KCs are oxidized from dietary yellow precursors, presumably by the avian carotenoid ketolase CYP2J19, the regulation and constraints of which have important implications for condition-dependence and honest signalling of carotenoid colour displays. We investigated hepatic *CYP2J19* gene expression in the seasonally and sexually dichromatic southern red bishop (*Euplectes orix*) in relation to season, sex, progression of the prenuptial moult, testis size, body condition, redness and circulating sex steroids. A coloration function of CYP2J19 is supported by a seasonal upregulation prior to and during the carotenoid-depositing stage of the male prenuptial moult. However, *CYP2J19* expression was similarly high in females (which do not moult prenuptially), and remained high in males after moult, suggesting additional or alternative roles of hepatic CYP2J19 or its products, such as detoxification or antioxidant functions. In males, the *CYP2J19* upregulation preceded and was unrelated to the rise in plasma testosterone, but was correlated with androstenedione, probably of adrenal origin and compatible with luteinizing hormone-induced and (in females) oestrogen-suppressed moult. Finally, contrary to ideas that carotenoid ketolation rate mediates honest signalling of male quality, *CYP2J19* expression was not related to plumage redness or male body condition.

## 1. Introduction

The view of conspicuous yellow and red carotenoid-based colours as condition-dependent and honest signals is a pervasive and popular topic of study in behavioural ecology. Because carotenoids have essential physiological functions in animals (e.g. as antioxidants and vitamin A precursors) and must be obtained through the diet, there are several potential limitations and allocation conflicts that could mediate signal honesty (reviewed by [1]). However, after three decades and hundreds of studies, predominately in birds [2,3], the empirical support for carotenoid-based honest signalling is thin, inconsistent and debated (e.g. [4–8]).

In recent years, the classic ‘resource allocation’ models of avian carotenoid honesty have given way to an emphasis on the metabolism of carotenoids [7,9], in particular the ability of several species to produce red ketocarotenoids (KCs) by C4-oxygenation (ketolation) of the yellow carotenoids that are common in avian diets [10–13]. There are several potential mechanisms by which the rate or efficiency of ketolation might link coloration to some phenotypic or genetic quality; for example, if the ketolation is energetically demanding, entails oxidative stress or is linked to ‘vital cellular processes' [14], such as vitamin A homeostasis [9], detoxification ability [15] or cellular respiration [16]. In the intriguing ‘shared-pathway hypothesis’ (SPH), Johnson & Hill [17] proposed that carotenoid ketolation occurs in the mitochondria and is so strongly linked to the electron transport chain that KC redness becomes an uncheatable ‘index signal’ (sensu [18]) of respiratory quality.

While these ideas have received some indirect support [19–22], the central assumption that carotenoid ketolation (rather than access, uptake, or transport) is the main limitation on redness has not been possible to directly explore until the recent identification of the gene *CYP2J19* [15,23], which putatively codes for the avian carotenoid ketolase. From a conserved role in the avian retina for oil droplet pigmentation, *CYP2J19* appears to have been co-opted for integumentary pigmentation in many lineages [24]. *CYP2J19* has been further implicated for plumage coloration in several weaverbird (Ploceidae) species [25], and recently also in the red-backed fairy wren (*Malurus melanocephalus*) [26] and red-fronted tinkerbird (*Pogoniulus pusillus*) [27].

CYP2J19 belongs to the diverse family of cytochrome P450 enzymes (CYP's hereafter) involved in endobiotic metabolism and oxidation of xenobiotics, in this case, the carotenoids. *CYP2J19* regulation and activity may thus provide a useful handle on the genetic and physiological control of red carotenoid coloration, and on the idea that CYP2J19 activity (or ‘catalytic rate’) is a major determinant of KC-based colour signal variation (redness). Since CYP's are primarily transcriptionally regulated [28–30] (yet post-transcriptional and post-translational regulation has been described especially for endobiotic networks such as steroid metabolism [30]), *CYP2J19* gene expression should be an important predictor of interspecific [25] and intraspecific variation in plumage redness.

**Figure 1.**
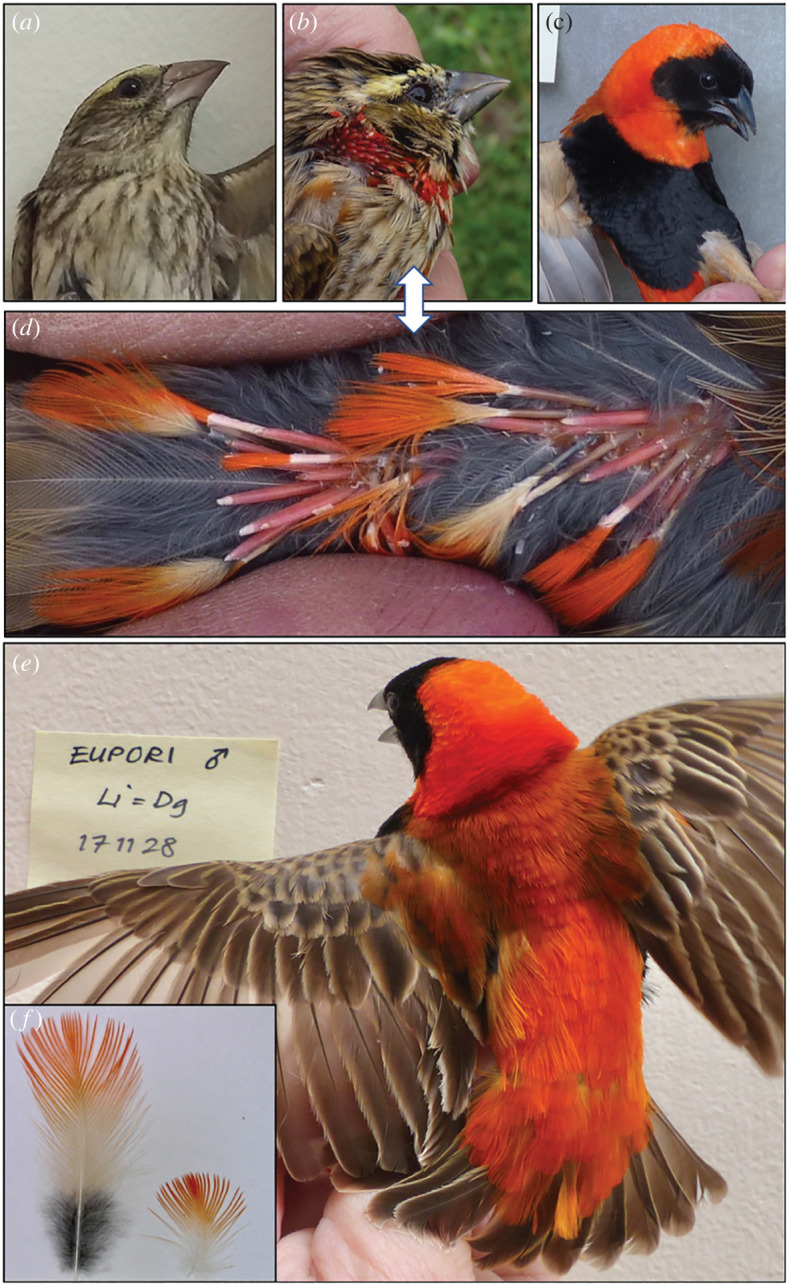
Prenuptial moult stages in male southern red bishops: CARD, before or during carotenoid deposition in feathers; PostCARD, after completed carotenoid deposition (see methods for detailed criteria). (*a*) CARD male in pre-breeding plumage just prior to moult start, still indistinguishable from female and non-breeding male plumage. (*b*) CARD male with ongoing carotenoid deposition in emerging collar feathers. (*c*) A fully nuptial PostCARD male. (*d*) Growing rump feathers, some with ongoing (pins or just emerged vane) and some with completed carotenoid deposition. (*e*) Fully nuptial plumage on a PostCARD male, with fully grown rump and collar feathers in the inset picture (*f*).

Here, for the first time (to our knowledge), we explore natural intraspecific variation in CYP2J19 activity, by analysing hepatic (liver) *CYP2J19* expression before and during development of the sexually and seasonally dichromatic red plumage of southern red bishops (*Euplectes orix*). Whereas female plumage is brown year-round and indistinguishable from that of non-breeding males breeding males sport one of the most spectacular carotenoid-based avian colour displays (figure 1*c,e*). The red coloration is well researched with respect to colour production mechanisms, social and sexual selection, and directional evolution from yellow ancestors [31–39]. The male breeding plumage (figure 1) is produced before the breeding season in a prenuptial moult, during which the emerging feather tips co-deposit KCs (e.g. *α*-doradexanthin and astaxanthin) along with their dietary precursors (lutein and zeaxanthin) [13,40]. In the comparative study of 16 Ploceids in breeding plumage that established the association between hepatic *CYP2J19* and KC-coloration [25], the red bishop had the second highest *CYP2J19* expression.

We examined the following issues with regard to seasonal, sexual and individual variation in hepatic *CYP2J19* expression: (i) functional confirmation, (ii) regulatory mechanisms, and (iii) role in honest signalling. We tested the assumption that integumentary coloration is the main function of hepatic *CYP2J19*, predicting male-biased expression timed with onset of the prenuptial moult, in particular, the initial moult stage during which carotenoids are deposited into emerging feathers (figure 1). We then explored potential regulatory mechanisms for *CYP2J19* expression, focusing on associations with reproductive readiness (i.e. testis size) and steroid hormones. The understanding of genetic versus hormonal control of carotenoid coloration lags behind that of melanin coloration [41–43], and is interesting with regard to the endocrine control of secondary sex traits and testosterone (T) mediated honest signalling [42,44]. Pioneering work on captive southern red bishops and other *Euplectes* species [45], although largely focused on luteinizing hormone (LH) dependence of melanin pigmentation, indicated stimulatory androgenic effects on carotenoid coloration. A relationship between T and carotenoid pigmentation has been confirmed for other species in recent years [43,46,47], and may be exerted in part via T-mediated *CYP2J19* expression [26].

Finally, we examined the potential role played by CYP2J19 in honest signalling, using a reflectance-based metric of hue (‘redness' hereafter), as the most reliable measure of feather carotenoid content [48]. As predicted by classic handicap (costly) signalling [44,49–52], as well as the index (cost-free) signalling [18] proposed by the SPH (see above), CYP2J19 activity (ketolation rate) should be a limiting factor for KC-pigmentation, resulting in a positive correlation between redness and *CYP2J19* expression. Furthermore, from the postulated dependence of carotenoid ketolation on cellular respiration and mitochondrial function, we also predicted positive covariation between *CYP2J19* expression and a common measure of body condition in birds (relative body mass).

## 2. Material and methods

### 2.1. Study species, field methods and sample collection

Adult southern red bishops were captured in 2013, 2016 and 2017 (*n* = 68) by morning mistnetting at communal night roosts and (subsequently) breeding sites in Kwazulu-Natal, South Africa. Netting occurred primarily in the Pietermaritzburg area (29°35' S, 30°26' E), but our sample size includes eight birds from Newcastle (27°44' S, 29°59' E). Most of the birds (57 males, six females) were caught and sampled at the start of the breeding (rainy) season, more precisely to span the male prenuptial moult period from *ca* mid-October to late November [53], but five birds (four males, one female) were caught in the non-breeding (dry) season (May–June). The growth, if any, of carotenoid-pigmented plumage was carefully inspected, and males were assigned to either of two prenuptial moult stages: (i) CARD males (*n* = 30) either without visible signs of moult (figure 1*a*) or with ongoing carotenoid deposition (figure 1*b*,*c*), the latter defined by having at least some entirely or proximally red feather ‘buds' (less than 3 mm), ‘pins' (greater than 3 mm but vane not emerging) or ‘vanes' (vane protruding max 5 mm); and (ii) PostCARD males (*n* = 27; figure 1*c*,*e*) in partial or complete nuptial plumage, in which all red feathers had grown past the initial carotenoid-depositing stage, i.e. no longer red at the base.

Standard morphometrics included body mass (±0.1 g) and tarsus length (±0.1 mm), from which a body condition measure was derived as the standardized residuals from a linear regression of ln(body mass) on 3*ln(tarsus length) [54]. Blood was drawn from the neck vein and exactly 100 µl was added to 1 ml acetone and stored at −20°C until further analysis. Liver (right lobe) samples were collected from freshly euthanized birds (via rapid decapitation), placed in DNA/RNA-shield (Zymo), incubated for 12 h at room temperature and then stored at −80°C until laboratory analyses. Left testicle length was used as a measurement for testis development (recrudescence) and an indicator of reproductive readiness (*n* = 23 CARD males, *n* = 27 PostCARD males with testis length measurements).

### 2.2. Reflectance spectrometry and colour metrics

If the male red breast patch was sufficiently developed (figure 1*c*; *n* = 26), its spectral reflectance was measured using equipment and methods previously described [34,48] and detailed in the electronic supplementary material. Following Andersson & Prager [48] and averaged from three replicate scans per bird, ‘redness' was computed as the objective hue metric *λ*_R50_ (the wavelength at which reflectance is halfway between its minimum and its maximum). For saturated pigmentary colours, hue (or ‘spectral location’ *sensu* [55]), is the only psychophysical colour metric consistently correlated with pigment concentration [48], and *λ*_R50_ is therefore the only colour measure analysed here. For further details and arguments, see Andersson [56] and Andersson & Prager [48].

### 2.3. Quantification of *CYP2J19* gene expression

Livers were homogenized using TissueLyser II (Qiagen), total RNA was extracted from homogenates using an RNeasy Plus Mini Kit (Qiagen) and DNase digestion was performed using an RNase-free DNase set (Qiagen). First-strand synthesis was performed with 10 µl RNA (100 ng µl^−1^) using an iScript cDNA Synthesis Kit (Bio-Rad), according to the manufacturer's instructions. Reactions were run in a MyCycler thermal cycler (Bio-Rad) under the following conditions: 5 min at 25°C, 30 min at 42°C and 5 min at 85°C. All complementary DNA (cDNA) samples were diluted to a final concentration of 25 ng µl^−1^.

Reverse transcription-quantitative polymerase chain reation (RT-qPCR) was performed in a CFX connect real-time PCR detection system (Bio-Rad) using 5 µl SsoAdvanced Universal SYBR Green Supermix, 0.5 µl of each primer (8 µM), and 4 µl cDNA (25 ng µl^−1^). Three control genes (β-actin, GAPDH and HPRT1) were run in separate plates. All primers were used in previous RT-qPCR work [15,25] and were purchased from Eurofins. Each sample, as well as positive and negative controls, were run in triplicate for each condition. Conditions for RT-qPCR were as follows: 3 min at 95°C, followed by 40 cycles of 10 s at 95°C and 30 s at 61°C (59°C for GAPDH) and a final melt curve. Normalization followed Pfaffl [57] using the mean of the three control genes.

### 2.4. Quantification of steroid hormones

In blood samples from 14 randomly selected CARD males and six PostCARD males, steroid titres of T, androstenedione (A4), dihydrotestosterone (DHT), oestrone (E1) and oestradiol (E2), were quantified using the gas chromatography-tandem mass spectrometry (GC-MS/MS) method of Ankarberg-Lindgren *et al*. [58]. Further details can be found in the electronic supplementary material. The remaining 46 samples were retained for future analyses of carotenoid content.

### 2.5. Statistical analyses

In order to determine the effect of seasonality (non-breeding versus breeding) on *CYP2J19* expression levels, we used a one-way ANOVA on the complete dataset. To test whether *CYP2J19* is differentially expressed in males and females, we used a one-way ANOVA on a dataset restricted to the breeding season. We then evaluated whether, during the breeding season, *CYP2J19* expression follows similar patterns as regards date and body condition in males and females, using a linear model with normalized *CYP2J19* expression (square root transformed) as a response variable and Julian date, body condition and their two-way interactions with sex as model predictors. To explore how *CYP2J19* expression is influenced by internal and external variables in males during the breeding season, we used a linear model, including normalized *CYP2J19* expression (square root) as a response variable and body condition, testis size and their two-way interactions with carotenoid deposition stage, as well as Julian date as covariates. To evaluate whether the hue of red breast feathers of full nuptial plumed males was predicted by *CYP2J19* expression, we used a linear model with hue as a response variable and normalized *CYP2J19* expression, body condition and Julian date as predictors. Finally, given the smaller sample sizes, we used Spearman rank correlations (rather than linear models) to determine the relationship between hormone levels, normalized *CYP2J19* expression and testis size. These initially included all males and were additionally conducted separately for CARD and PostCARD males when the first correlation was statistically significant. All tests were performed using R (v. 3.5.2 [59]). Covariates specified in interaction terms were scaled to allow interpretation of their main effects [60]. In multi-factor models, we performed model simplification using the automated *step* function (package *stats*) with backward and forward procedures to eliminate non-significant terms from the maximal models. Diagnostic plots were used for model validation [61].

## 3. Results

### 3.1. Seasonal but not sexual differences in *CYP2J19* expression

**Figure 2.**
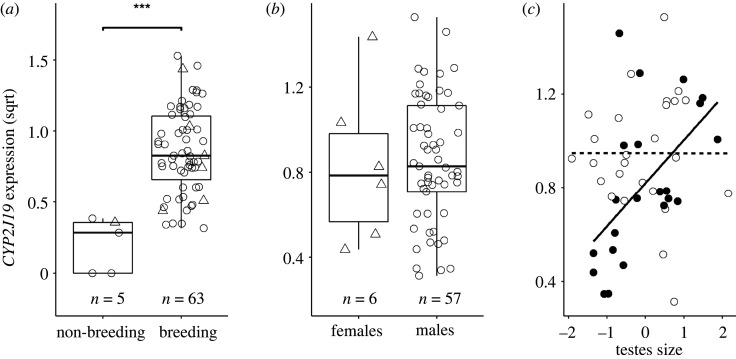
*CYP2J19* expression in relation to (*a*) season, (*b*) sex (during the breeding season), and (*c*) reproductive development. (*a*,*b*) Raw values are represented by circles (males) or triangles (females). Boxplots show median, first and third quantiles with whiskers extending no further than 1.5 times the inter-quartile range. Horizontal jitter was added to improve visualization of individual data points. (*c*) Raw values are represented by black (CARD) or white (PostCARD) circles. Lines represent the linear model fit for CARD (solid line) and PostCARD (dashed line) males. Testis length was scaled within each prenuptial moult stage, to allow comparison between categories (see the electronic supplementary material, figure S1 for an unscaled version of the plot). In all plots, normalized *CYP2J19* expression values are square root transformed. ***p < 0.001.

Compared with birds sampled in the non-breeding season (five males, one female), the birds caught in the early breeding season (57 males, six females) had significantly higher *CYP2J19* expression (ANOVA: *F*_1,66_ = 22.74, *p* < 0.001; figure 2*a*). *CYP2J19* expression did not, however, differ between males and females during this period (ANOVA: *F*_1,61_ = 0.05, *p* = 0.819; figure 2*b*), and was not related to Julian date in either males or females in subsequent models (electronic supplementary material, tables S1 and S2).

### 3.2. *CYP2J19* expression and body condition

In a model assessing the relationship between *CYP2J19* and body condition, there was a strong interaction with sex (*F*_1,52_ = 10.30, *p* = 0.002; electronic supplementary material, table S1) owing to a negative trend in females (*n* = 6), and a positive relationship in males (*n* = 50). Although the bivariate relationship in males was significant (Spearman *r*_s_ = 0.31, *n* = 50, *p* = 0.025), condition had no partial effect in the subsequent model with other predictors, in particular testis size (see below and the electronic supplementary material, table S2) with which body condition was strongly correlated (Spearman *r*_s_ = 0.46, *n* = 50, *p* < 0.001).

### 3.3. Male *CYP2J19* expression varies with testis size and moult stage

Examination of individual variation in *CYP2J19* expression of males during the breeding season revealed a significant interaction between testis size and carotenoid deposition stage: testis size positively correlated with *CYP2J19* during the CARD stage, but did not predict *CYP2J19* in the PostCARD stage (linear model: estimate = −0.20, s.e. = 0.11, *F*_1,44_ = 5.14, *p* = 0.028; electronic supplementary material, table S2, figure S1; figure 2*c*). This model also revealed an overall positive relationship between *CYP2J19* expression and testis size (linear model: estimate = 0.21, s.e. = 0.09, *F*_1,44_ = 4.29, *p* = 0.044; electronic supplementary material, table S2) and a trend for higher expression levels in PostCARD than CARD males (linear model: estimate = 0.18, s.e. = 0.12, *F*_1,44_ = 3.30, *p* = 0.076; electronic supplementary material, table S2).

### 3.4. *CYP2J19* expression is unrelated to plumage redness

Hepatic *CYP2J19* expression did not predict the redness (hue) of the breast patch in PostCARD males (linear model: estimate = 2.11, s.e. = 2.48, *F*_1,22_ = 5.31, *p* = 0.4 in the full model). The only significant predictor of redness was a negative effect of Julian date (linear model: estimate = −0.21, s.e. = 0.09, *F*_1,23_ = 6.06, *p* = 0.022), but an almost significant negative effect of body condition (linear model: estimate = −33.57, s.e. = 17.08, *F*_1,23_ = 3.43, *p* = 0.077) was also maintained in the final model.

### 3.5. *CYP2J19* expression and steroid hormones

**Figure 3.**
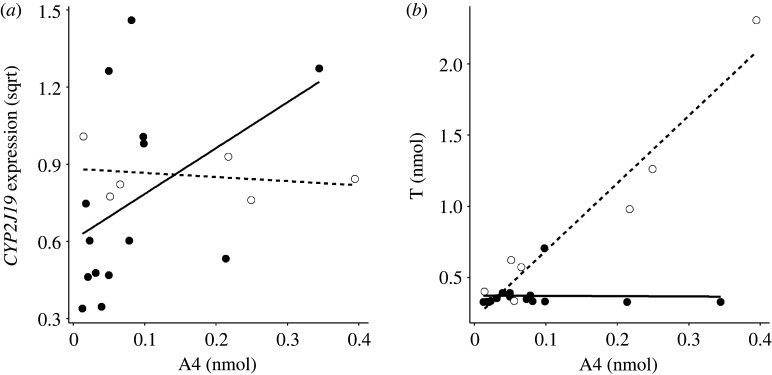
Relationship between androstenedione (A4) titre and (*a*) *CYP2J19* expression and (*b*) testosterone (T) titre. Raw values are represented by black (CARD) or white (PostCARD) circles. Lines represent least-squares regressions for CARD (solid line) and PostCARD (dashed line) males. Normalized *CYP2J19* expression is square root transformed.

Relationships with steroid hormones were explored with Spearman correlations (electronic supplementary material, tables S3 and S4). *CYP2J19* expression was positively correlated with A4 levels (all males: *n* = 20, *r*_s_ = 0.466, *p* = 0.040; electronic supplementary material, table S3). This relationship appears to be driven by the males undergoing carotenoid deposition into feathers (CARD: *n* = 14, *r*_s_ = 0.581, *p* = 0.029; PostCARD: *n* = 6, *r*_s_ = −0.314, *p* = 0.564; figure 3*a*; electronic supplementary material, table S3). None of the other androgenic (T and DHT) or oestrogenic (E1 and E2) hormones were correlated with *CYP2J19* expression when all males were pooled (T: *r*_s_ = 0.287; DHT: *r*_s_ = 0.313; E1: *r*_s_ = 0.214; E2: *r*_s_ = −0.192; all *p* > 0.2; electronic supplementary material, table S3). Although there was no overall correlation between A4 and T (all males: *n* = 22, *r*_s_= 0.303, *p* = 0.170), a positive relationship between the two androgens was identified in PostCARD males (*n* = 15, *r*_s_ = 0.821, *p* = 0.034; figure 3*b*), but not in CARD males (*n* = 7, *r*_s_ = 0.061, *p* = 0.832; figure 3*b*). Finally, T, DHT and E1, but not A4 or E2, were positively associated with testis size (T: *r*_s_ = 0.678, *p* = 0.003; DHT: *r*_s_ = 0.538, *p* = 0.026; E1: *r*_s_ = 0.553, *p* = 0.021; A4: *r*_s_ = 0.248, *p* = 0.338; E2: *r*_s_ = 0.143, *p* = 0.583; electronic supplementary material, table S4). However, when assessed within CARD and PostCARD males separately, none of these hormones significantly correlated with testis size (all *p* > 0.2).

## 4. Discussion

We uncover, for the first time, to our knowledge, seasonal and sexual variation in hepatic expression of the putative avian carotenoid ketolase gene *CYP2J19* and, in particular, individual variation during development of an extreme KC-based colour signal. We found that *CYP2J19* expression was higher during the breeding compared to the non-breeding season (at least in males, strictly speaking, since only one female was sampled in the non-breeding season). Somewhat surprisingly given the exclusively male KC pigmentation, *CYP2J19* expression was not higher in males than in females during the breeding season and was unrelated to plumage redness (hue), contrary to the advocated roles of CYP2J19 in condition-dependent and honest signalling. Regarding regulatory mechanisms, in the carotenoid-depositing stage of the prenuptial moult, *CYP2J19* expression correlated with testicular growth, but was unrelated to plasma T. Instead, only A4 positively covaried with *CYP2J19* expression. In the light of these findings, we discuss *CYP2J19* with regard to function, regulation and potential role in honest signalling.

### 4.1. Function of CYP2J19

Although CYP2J19 still awaits functional (i.e. experimental) confirmation, the higher hepatic expression in red compared to yellow species [25], together with the upregulation prior to the male prenuptial moult indicated in this study, strongly support a role of *CYP2J19* behind the KC-pigmentation in southern red bishops and other red species (except those with obvious dietary sources of KC-pigments). However, the similar expression levels in females (which have neither carotenoid coloration nor prenuptial moult), and the high expression in males after moult, suggest that hepatic CYP2J19 itself, or its products (KCs), have functions other than pigmentation. One possibility is that, in response to the increased dietary carotenoid ingestion during the rainy season, the original and still major role of CYP2J19 may be detoxification, by conversion of pro-oxidant xanthophylls such as lutein [62] into less harmful and/or more excretable KCs.

Alternatively, or in addition, while the ‘classic' antioxidant, pro-vitamin or immune-stimulating functions of carotenoids (e.g. [1]) have been questioned or dismissed as regards dietary xanthophylls [4,6,63,64], either of these functions may apply to some or all KCs, in particular during breeding which typically entails severe oxidative stress from demanding behaviours [65,66], as well as gamete production [67]. In females, allocation of carotenoids to the eggs [68] has been shown to influence chick immune function [69], survival and even adult carotenoid coloration [70]. In some gulls, KCs are preferentially deposited in egg yolk over the hydroxy-carotenoids (lutein, zeaxanthin) [71], which may indicate unique benefits of yolk KCs. Considering the red colour of red bishop egg yolks (S. Andersson 2022, personal observation), yolk deposition of KCs seems likely and the high female *CYP2J19* expression might thus be a physiological preparation for egg-laying.

Interestingly in this context, female *CYP2J19* expression appeared to be negatively related to body condition, as opposed to the positive bivariate relationship in males (which disappeared when controlling for testicular development). This could indicate a cost or trade-off unique to females, but given the small sample size (*n* = 6) and a spurious interaction with Julian date, we refrain from speculations and highlight the need for further studies on female-specific functions of CYP2J19 and KCs.

### 4.2. Regulatory mechanisms of *CYP2J19* expression

Given that the evolution of red KC-coloration is associated with co-option of *CYP2J19* expression to a new tissue, rather than with functional (coding) changes [25], some of the first questions to address concern the regulatory mechanisms; our results provide some first indications of the environmental, hormonal and sexual control of *CYP2J19*.

Firstly, as implied above, one might expect that *CYP2J19* is directly or indirectly regulated by the dietary uptake of its substrates (i.e. the KC precursors), as is the case of the carotenoid oxidases in the retinoid (vitamin A) pathway [72] and many other xenobiotic-induced liver CYP's [73]. We suggest that increased access to carotenoids from germinating grasses [74] at the start of the rainy season is a probable trigger of the significant seasonal upregulation of *CYP2J19* in males (and probably also females although represented by a single non-breeding individual). The regulation might be either via ligand-activation of nuclear receptors, as in many ‘xeno-sensing' CYP's [75], or via the hypothalamic-pituitar-adrenal axis and adrenal steroids (A4) as discussed below. Evidence compatible with such dietary regulation of *CYP2J19* comes from the KC-pigmented house finch (*Carpodacus mexicanus*) [76] and common crossbill (*Loxia curvirostra*) [77,78], in which increased plasma hue and carotenoid content, respectively, coincided with a shift to carotenoid-rich diets one to two months before the moult period. Moreover, in the crossbills, there was a one-month delay between increased plasma concentrations of dietary carotenoids and the rise in KCs [77], suggesting a lag in either production or mobilization (or both) of KC's. Early upregulation of *CYP2J19* may thus be an adaptation to begin accumulating KCs well before the brief time-window of plumage deposition in males (and yolk deposition in females). In terms of signal content and honesty, this differs markedly from KC-coloured bare parts (skin, bill) in which redness may dynamically reflect either circulating carotenoid levels (but see [79]) or, in the case of ‘peripheral converters', integumentary *CYP2J19* activity. Further research examining covariation between hepatic *CYP2J19*, circulating carotenoids and seasonal availability of carotenoid precursors is needed in order to determine the importance of diet in driving individual variation in *CYP2J19* expression.

Second, *CYP2J19* expression may be under hormonal control. The hormonal control of avian carotenoid coloration is poorly known, but our study species (*E. orix*) is an excellent place to start given that its close relative, the northern red bishop (*Euplectes franciscanus*), was the main subject of the foundational work by Witschi [45,80]. His work established that male nuptial moult and plumage pigmentation are not induced by T, but rather by the hypophyseal LH, although some influence of androgens on ‘carotenoid mobilization' was also suggested. In recent years, and largely owing to the interest in T-mediated ‘honest signalling' (see below), T-effects on carotenoid coloration have been indicated in some species with bare-part (bill, skin) pigmentation [43,81,82], and in one species with KC-based plumage ornamentation; the red-backed fairy wren (*M. melanocephalus)* [46,47]. Intriguingly, in this species, Khalil *et al.* [26] found that exogeneous T (implants) resulted in increased hepatic expression of *CYP2J19*, albeit in a very small sample (*n* = 3).

By contrast, in the southern red bishop, Edler & Friedl [31] found no relationship between plasma T and redness, and the present study found no signs of T-regulation of *CYP2J19* since gene expression was (i) high in females as well as in males with regressed or at most half-grown testes (less than 5 mm), and (ii) unrelated to circulating T, which was elevated only in PostCARD males with large or fully grown testis (5–13 mm). In fact, the highest *CYP2J19* expression levels were found in two brown males with no signs of moult, fully regressed testes and the lowest recorded levels of T (*ca* 0.3 ng l^−1^). To conclude, if there is an influence of T on plumage redness in this species, it is probably not via CYP2J19 activity and KC production and more likely owing to the ‘carotenoid mobilization' that Witschi [80] suggested, perhaps via the uptake and lipoprotein transport mechanisms elegantly revealed by McGraw *et al.* [43].

Interestingly, however, another steroid, A4 had a positive association with *CYP2J19* expression in KC-depositing (CARD) males. A4 is a precursor of both T and oestrogenic steroids and may therefore be secreted along with its metabolite from the gonads, but since the testes were small and there was no relationship between A4 and the low T-levels during the CARD stage, A4 was probably not gonadal at this time (but may be so in PostCARD males where indeed A4 was positively correlated with the rising T-levels; see figure 3*b*). Instead, we suggest that the A4 in CARD males may be produced in the adrenals, as in several bird species [83–86]. Adrenal A4 and/or its precursor dehydroepiandrosterone (DHEA) may regulate hepatic *CYP2J19* expression either directly or, like the neuroendocrinological control of aggression by DHEA when gonadal T is low or absent (e.g. [87]), after peripheral conversion to T or E2. Since adrenal steroidogenesis is partly under the control of LH (e.g. in humans and mice [85,88]), adrenal A4 could be an early hypothalamic induced and pre-gonadal trigger of *CYP2J19* and KC accumulation. This is consistent with the described earlier production of red compared to black feathers during moult and compatible with the LH-induction of full prenuptial moult and melanin pigmentation demonstrated by Witschi [45,80] in the closely related northern red bishop (*E. franciscanus*).

### 4.3. Implications for honest signalling

The widely held view that carotenoid displays advertise health or ‘quality' is surprisingly unsubstantiated [1,8], especially in natural populations, and even where associations seem strong between carotenoid coloration and aspects of health or viability (e.g. [7,89,90]), the mechanistic links are debated. Most attended to in recent years is the SPH [14], which proposes that it is neither access nor allocation, but rather the metabolism of carotenoids, that prevents cheating, more specifically that carotenoid ketolation hinges directly on respiratory (i.e. mitochondrial) efficiency [16].

Here, by analysing *CYP2J19* expression in wild birds, during the production of an exaggerated and sexually selected KC-display, we address the core tenet of SPH, namely that carotenoid ketolation rate is the limitation and link between KC-based redness and condition. To the contrary, we found that plumage redness (hue) was unrelated to *CYP2J19* expression and that neither was predicted by body condition, a reliable health indicator which, in the congeneric widowbirds, is associated with their long tails [39,91,92] as well as with their elaborate behavioural displays [54]. Compared to such obviously costly traits, the proposed ‘energetic cost of carotenoid utilization' [16,93] seems likely to be minor, even in such a formidably carotenoid-coloured species as the southern red bishop, and thus unlikely to mediate honest signalling. In addition, if the catalytic rate of CYP2J19 was a major source of redness variation, one would expect a positive relationship between *CYP2J19* expression and redness, which we did not find. Although hue could only be investigated in males with quantifiable colour patches, i.e. one to three weeks after pigment deposition, gene expression levels did not decline after moult, which is why it seems reasonable to assume that individual expression levels, or at least their rank order, did not change. Moreover, that *CYP2J19* expression showed no signs of downregulation after moult also in itself speaks against carotenoid ketolation as a costly and (as argued above) primarily signal-producing trait.

Here should also be mentioned the special case of the SPH which posits that carotenoid ketolation takes place in the mitochondria and shares essential machinery with cellular respiration, thereby creating an inexorable and ‘cost-free' link between coloration and individual quality. This idea was proposed by Johnson & Hill [17] and recently labelled the inner mitochondrial membrane carotenoid oxidation hypothesis by Cantarero *et al*. [20]. While ‘cost-free’ ketolation in principle could explain the lack of sex-biased expression as well as the lack of condition-dependence, the absent correlation between coloration and *CYP2J19* expression (inasmuch as it reflects ketolation activity) argues against also this version of the SPH. Moreover, the critical assumption that ketolation occurs exclusively in the mitochondria is not well founded and in fact rather unlikely given that the vast majority of CYP's (e.g. 55 of 57 human CYP's) locate to the endoplasmatic reticulum, which in the house finch indeed appeared to have higher KC concentration than the mitochondria [22].

Finally, we find no support for T-mediated honesty of carotenoid coloration (e.g. [42]) in southern red bishops. T-levels were unrelated to both redness and *CYP2J19* expression and, most importantly, *CYP2J19* upregulation as well as carotenoid deposition in feathers occurred before testes were developed and plasma T-levels began to rise. Any influence of T on redness, via effects on carotenoid uptake, conversion or transport [43], would thus be driven by the low pre-gonadal T levels, which in addition seem unlikely to have an immuno-suppressive effect (the core assumption of the immunocompetence handicap hypothesis).

### 4.4. Concluding remarks

Based on its elevated expression before and during the male prenuptial moult in southern red bishops, hepatic CYP2J19-catalysed carotenoid ketolation is most likely a necessary component of the red coloration. However, ketolation rate seems to be neither condition-dependent nor a significant source of individual redness variation, contrary to current ideas that carotenoid conversion efficiency reliably links redness to individual quality. Similar expression levels in females, and sustained levels in males after moult, suggest other roles of hepatic CYP2J19 and KC's, such as detoxification or antioxidant functions. As regards regulation, *CYP2J19* expression preceded testicular growth and was independent of plasma T, but positively correlated with A4. This might be of adrenal origin and compatible with the LH-triggered nuptial pigmentation previously shown in this genus, opening the door for exciting research into the regulatory mechanisms of CYP2J19 at both physiological and molecular levels.

## Data Availability

Data and code used in the work can be found in the Dryad repository: https://doi.org/10.5061/dryad.ht76hdrjg [94]. Data is also provided in the electronic supplementary material [95].

## References

[1] Svensson PA, Wong B. 2011 Carotenoid-based signals in behavioural ecology: a review. Behaviour **148**, 131-189. (10.1163/000579510X548673)

[2] Hill GE, McGraw KJ. 2006 Bird coloration volume I: mechanisms and measurements. Cambridge, MA: Harvard University Press.

[3] Hill GE, McGraw KJ. 2006 Bird coloration volume II: function and evolution. Cambridge, MA: Harvard University Press.

[4] Costantini D, Møller A. 2008 Carotenoids are minor antioxidants for birds. Funct. Ecol. **22**, 367-370. (10.1111/j.1365-2435.2007.01366.x)

[5] Lozano GA. 2001 Carotenoids, immunity, and sexual selection: comparing apples and oranges? Am. Nat. **158**, 200-203. (10.1086/321313) 18707348

[6] Simons MJ, Cohen AA, Verhulst S. 2012 What does carotenoid-dependent coloration tell? Plasma carotenoid level signals immunocompetence and oxidative stress state in birds–a meta-analysis. PLoS ONE **7**, e43088. (10.1371/journal.pone.0043088) 22905205 PMC3419220

[7] Weaver RJ, Santos ES, Tucker AM, Wilson AE, Hill GE. 2018 Carotenoid metabolism strengthens the link between feather coloration and individual quality. Nat. Commun. **9**, 1-9. (10.1038/s41467-017-02649-z) 29311592 PMC5758789

[8] Koch RE, Hill GE. 2018 Do carotenoid-based ornaments entail resource trade-offs? An evaluation of theory and data. Funct. Ecol. **32**, 1908-1920. (10.1111/1365-2435.13122)

[9] Hill GE, Johnson JD. 2012 The vitamin A-redox hypothesis: a biochemical basis for honest signaling via carotenoid pigmentation. Am. Nat. **180**, E127-E150. (10.1086/667861) 23070328

[10] Brush AH. 1990 Metabolism of carotenoid pigments in birds. FASEB J. **4**, 2969-2977. (10.1096/fasebj.4.12.2394316) 2394316

[11] Hill GE. 1996 Redness as a measure of the production cost of ornamental coloration. Ethol. Ecol. Evol. **8**, 157-175. (10.1080/08927014.1996.9522926)

[12] McGraw KJ. 2006 The mechanics of carotenoid-based coloration in birds. In Bird coloration, vol 1 mechanisms and measurements (eds G.E. Hill, K.J. McGraw), pp. 177-242. Cambridge, UK: Harvard University Press.

[13] Prager M, Johansson EA, Andersson S. 2009 Differential ability of carotenoid C4-oxygenation in yellow and red bishop species (*Euplectes* spp.). Comp. Biochem. Physiol. B: Biochem. Mol. Biol. **154**, 373-380. (10.1016/j.cbpb.2009.06.015) 19686862

[14] Hill GE. 2011 Condition-dependent traits as signals of the functionality of vital cellular processes. Ecol. Lett. **14**, 625-634. (10.1111/j.1461-0248.2011.01622.x) 21518211

[15] Mundy NI et al. 2016 Red carotenoid coloration in the zebra finch is controlled by a cytochrome P450 gene cluster. Curr. Biol. **26**, 1435-1440. (10.1016/j.cub.2016.04.047) 27212402

[16] Hill GE. 2014 Cellular respiration: the nexus of stress, condition, and ornamentation. Integr. Comp. Biol. **54**, 645-657. (10.1093/icb/icu029) 24791751

[17] Johnson JD, Hill GE. 2013 Is carotenoid ornamentation linked to the inner mitochondria membrane potential? A hypothesis for the maintenance of signal honesty. Biochimie **95**, 436-444. (10.1016/j.biochi.2012.10.021) 23131590

[18] Smith JM, Harper D. 2003 Animal signals. Oxford, UK: Oxford University Press.

[19] Cantarero A, Alonso-Alvarez C. 2017 Mitochondria-targeted molecules determine the redness of the zebra finch bill. Biol. Lett. **13**, 20170455. (10.1098/rsbl.2017.0455) 29070589 PMC5665772

[20] Cantarero A, Mateo R, Camarero PR, Alonso D, Fernandez-Eslava B, Alonso-Alvarez C. 2020 Testing the shared-pathway hypothesis in the carotenoid-based coloration of red crossbills. Evolution **74**, 2348-2364. (10.1111/evo.14073) 32749066

[21] Cantarero A, Andrade P, Carneiro M, Moreno-Borrallo A, Alonso-Alvarez C. 2020 Testing the carotenoid-based sexual signalling mechanism by altering *CYP2J19* gene expression and colour in a bird species. Proc. R. Soc. B **287**, 20201067. (10.1098/rspb.2020.1067) PMC7735286 33171089

[22] Hill GE et al. 2019 Plumage redness signals mitochondrial function in the house finch. Proc. R. Soc. B **286**, 20191354. (10.1098/rspb.2019.1354) PMC6784716 31551059

[23] Lopes RJ et al. 2016 Genetic basis for red coloration in birds. Curr. Biol. **26**, 1427-1434. (10.1016/j.cub.2016.03.076) 27212400 PMC5125026

[24] Twyman H, Valenzuela N, Literman R, Andersson S, Mundy NI. 2016 Seeing red to being red: conserved genetic mechanism for red cone oil droplets and co-option for red coloration in birds and turtles. Proc. R. Soc. B **283**, 20161208. (10.1098/rspb.2016.1208) PMC5013772 27488652

[25] Twyman H, Prager M, Mundy NI, Andersson S. 2018 Expression of a carotenoid-modifying gene and evolution of red coloration in weaverbirds (Ploceidae). Mol. Ecol. **27**, 449-458. (10.1111/mec.14451) 29230900

[26] Khalil S, Welklin JF, McGraw KJ, Boersma J, Schwabl H, Webster MS, Karubian J. 2020 Testosterone regulates CYP2J19-linked carotenoid signal expression in male red-backed fairywrens (*Malurus melanocephalus*). Proc. R. Soc. B **287**, 20201687. (10.1098/rspb.2020.1687) PMC7542802 32933448

[27] Kirschel AN, Nwankwo EC, Pierce DK, Lukhele SM, Moysi M, Ogolowa BO, Hayes SC, Monadjem A, Brelsford A. 2020 CYP2J19 mediates carotenoid colour introgression across a natural avian hybrid zone. Mol. Ecol. **29**, 4970-4984. (10.1111/mec.15691) 33058329

[28] Tracy TS et al. 2016 Interindividual variability in cytochrome P450–mediated drug metabolism. Drug Metab. Dispos. **44**, 343-351. (10.1124/dmd.115.067900) 26681736 PMC4767386

[29] Rosvall KA, Burns CMB, Jayaratna SP, Ketterson ED. 2016 Divergence along the gonadal steroidogenic pathway: implications for hormone-mediated phenotypic evolution. Horm. Behav. **84**, 1-8. (10.1016/j.yhbeh.2016.05.015) 27206546 PMC4996689

[30] Tralau T, Luch A. 2013 The evolution of our understanding of endo-xenobiotic crosstalk and cytochrome P450 regulation and the therapeutic implications. Expert Opin. Drug Metab. Toxicol. **9**, 1541-1554. (10.1517/17425255.2013.828692) 23941336

[31] Edler AU, Friedl TW. 2010 Plumage colouration, age, testosterone and dominance in male red bishops (*Euplectes orix*): a laboratory experiment. Ethology **116**, 806-820.

[32] Edler AU, Friedl TW. 2012 Age-related variation in carotenoid-based plumage ornaments of male red bishops *Euplectes orix*. J. Ornithol. **153**, 413-420. (10.1007/s10336-011-0757-3)

[33] Edler AU, Friedl TW. 2011 Carotenoid-based plumage colouration in red bishops (*Euplectes orix*)—signalling presence rather than quality? Behaviour **148**, 1372-1392. (10.1163/000579511X608693)

[34] Ninnes CE, Webb SL, Andersson S. 2017 Are red bishops red enough? On the persistence of a generalized receiver bias in *Euplectes*. Behav. Ecol. **28**, 117-122. (10.1093/beheco/arw136)

[35] Ninnes CE, Adrion M, Edelaar P, Tella JL, Andersson S. 2015 A receiver bias for red predates the convergent evolution of red color in widowbirds and bishops. Behav. Ecol. **26**, 1212-1218. (10.1093/beheco/arv068)

[36] Ninnes C, Andersson S. 2014 Male receiver bias for red agonistic signalling in a yellow-signalling widowbird: a field experiment. Proc. R. Soc. B **281**, 20140971 (10.1098/rspb.2014.0971) PMC4123703 25056624

[37] Prager M, Andersson S. 2010 Convergent evolution of red carotenoid coloration in widowbirds and bishops (*Euplectes* spp.). Evol. Int. J. Organic Evol. **64**, 3609-3619. (10.1111/j.1558-5646.2010.01081.x) 20629731

[38] Pryke SR, Andersson S, Lawes MJ, Piper SE. 2002 Carotenoid status signaling in captive and wild red-collared widowbirds: independent effects of badge size and color. Behav. Ecol. **13**, 622-631. (10.1093/beheco/13.5.622)

[39] Pryke SR, Andersson S. 2003 Carotenoid-based epaulettes reveal male competitive ability: experiments with resident and floater red-shouldered widowbirds. Anim. Behav. **66**, 217-224. (10.1006/anbe.2003.2193)

[40] Andersson S, Prager M, Johansson EA. 2007 Carotenoid content and reflectance of yellow and red nuptial plumages in widowbirds (*Euplectes* spp.). Funct. Ecol. **21**, 272-281. (10.1111/j.1365-2435.2007.01233.x)

[41] Kimball RT. 2006 Hormonal control of avian coloration. In Bird coloration, vol 1: mechanisms and measurements (eds G.E. Hill, K.J McGraw), pp. 591-644. Cambridge, MA: Harvard University Press.

[42] Peters A. 2007 Testosterone and carotenoids: an integrated view of trade-offs between immunity and sexual signalling. Bioessays **29**, 427-430. (10.1002/bies.20563) 17450573

[43] McGraw KJ, Correa SM, Adkins-Regan E. 2006 Testosterone upregulates lipoprotein status to control sexual attractiveness in a colorful songbird. Behav. Ecol. Sociobiol. **60**, 117-122. (10.1007/s00265-005-0135-3)

[44] Folstad I, Karter AJ. 1992 Parasites bright males and the immunocompetence handicap. Am. Nat. **139**, 603-622. (10.1086/285346)

[45] Witschi E. 1935 Seasonal sex characters in birds and their hormonal control. Wilson Bull. **17**, 177-188.

[46] Lindsay WR, Webster MS, Schwabl H. 2011 Sexually selected male plumage color is testosterone dependent in a tropical passerine bird, the red-backed fairy-wren (*Malurus melanocephalus*). PLos ONE **6**, e26067 (10.1371/journal.pone.0026067) 21998753 PMC3187837

[47] Lindsay WR, Barron DG, Webster MS, Schwabl H. 2016 Testosterone activates sexual dimorphism including male-typical carotenoid but not melanin plumage pigmentation in a female bird. J. Exp. Biol. **219**, 3091-3099. (10.1242/jeb.135384) 27707865

[48] Andersson S, Prager M. 2006 Quantifying colors. In Bird coloration, vol 1: mechanisms and measurements (eds G.E. Hill, K.J McGraw), pp. 41-89. Cambridge, MA: Harvard University Press.

[49] Andersson M. 1986 Evolution of condition-dependent sex ornaments and mating preferences: sexual selection based on viability differences. Evolution **40**, 804-816. (10.1111/j.1558-5646.1986.tb00540.x) 28556175

[50] Grafen A. 1990 Sexual selection unhandicapped by the Fisher process. J. Theor. Biol. **144**, 473-516. (10.1016/S0022-5193(05)80087-6) 2402152

[51] Hamilton WD, Zuk M. 1982 Heritable true fitness and bright birds: a role for parasites? Science **218**, 384-387. (10.1126/science.7123238) 7123238

[52] Zahavi A. 1975 Mate selection: a selection for a handicap. J. Theor. Biol. **53**, 205-214. (10.1016/0022-5193(75)90111-3) 1195756

[53] Craig A. 1982 The breeding season of the red bishop. Ostrich **53**, 112-114. (10.1080/00306525.1982.9634735)

[54] Andersson S. 1994 Costs of sexual advertising in the lekking Jackson's widowbird. The Condor **96**, 1-10. (10.2307/1369058)

[55] Hailman JP. 1977 Optical signals: animal communication and light. Bloomington, IN: Indiana University Press.

[56] Andersson S. 2000 Efficacy and content in avian colour signals. In Animal signals: signalling and signal design in animal communication (eds Y. Espmark, T. Amundsen, G Rosenqvist), pp. 47-60. Oslo, Norway: Tapir Publishers.

[57] Pfaffl MW. 2001 A new mathematical model for relative quantification in real-time RT-PCR. Nucleic Acids Res. **29**, 2002-2007. 10.1093/nar/29.9.e45 PMC55695 11328886

[58] Ankarberg-Lindgren C, Dahlgren J, Andersson MX. 2018 High-sensitivity quantification of serum androstenedione, testosterone, dihydrotestosterone, estrone and estradiol by gas chromatography–tandem mass spectrometry with sex-and puberty-specific reference intervals. J. Steroid Biochem. Mol. Biol. **183**, 116-124. (10.1016/j.jsbmb.2018.06.005) 29894754

[59] R Core Team. 2016.*R: a language and environment for statistical computing*. Vienna, Austria: R Foundation for Statistical Computing.

[60] Schielzeth H. 2010 Simple means to improve the interpretability of regression coefficients. Methods Ecol. Evol. **1**, 103-113. (10.1111/j.2041-210X.2010.00012.x)

[61] Zuur AF, Ieno EN. 2016 A protocol for conducting and presenting results of regression-type analyses. Methods Ecol. Evol. **7**, 636-645. (10.1111/2041-210X.12577)

[62] Amengual J, Lobo GP, Golczak M, Li HNM, Klimova T, Hoppel CL, Wyss A, Palczewski K, Von Lintig J. 2011 A mitochondrial enzyme degrades carotenoids and protects against oxidative stress. FASEB J. **25**, 948-959. (10.1096/fj.10-173906) 21106934 PMC3042841

[63] Isaksson C, Andersson S. 2008 Oxidative stress does not influence carotenoid mobilization and plumage pigmentation. Proc. R. Soc. B **275**, 309-314. (10.1098/rspb.2007.1474) PMC2593728 18029305

[64] Koch RE, Staley M, Kavazis AN, Hasselquist D, Toomey MB, Hill GE. 2019 Testing the resource trade-off hypothesis for carotenoid-based signal honesty using genetic variants of the domestic canary. J. Exp. Biol. **222**, jeb188102. (10.1242/jeb.188102) 30877227

[65] Swanson DL. 2010 Seasonal metabolic variation in birds: functional and mechanistic correlates. In Current ornithology volume 17 (ed. CF Thompson), pp. 75-129. Berlin, Germany: Springer.

[66] Tieleman BI, Versteegh MA, Fries A, Helm B, Dingemanse NJ, Gibbs HL, Williams JB. 2009 Genetic modulation of energy metabolism in birds through mitochondrial function. Proc. R. Soc. B **276**, 1685-1693. (10.1098/rspb.2008.1946) PMC2660998 19324832

[67] Hayward A, Gillooly JF. 2011 The cost of sex: quantifying energetic investment in gamete production by males and females. PLos ONE **6**, e16557 (10.1371/journal.pone.0016557) 21283632 PMC3026017

[68] Saino N, Bertacche V, Ferrari Raffaella P, Martinelli R, Møller Anders P, Stradi R. 2002 Carotenoid concentration in barn swallow eggs is influenced by laying order, maternal infection and paternal ornamentation. Proc. R. Soc. Lond. B **269**, 1729-1733. (10.1098/rspb.2002.2088) PMC1691081 12204135

[69] Saino N, Ferrari R, Romano M, Martinelli R, Møller AP. 2003 Experimental manipulation of egg carotenoids affects immunity of barn swallow nestlings. Proc. R. Soc. Lond. B **270**, 2485-2489. (10.1098/rspb.2003.2534) PMC1691538 14667340

[70] McGraw K, Adkins-Regan E, Parker R. 2005 Maternally derived carotenoid pigments affect offspring survival, sex ratio, and sexual attractiveness in a colorful songbird. Naturwissenschaften **92**, 375-380. (10.1007/s00114-005-0003-z) 16049690

[71] Blount J, Surai P, Houston D, Møller A. 2002 Patterns of yolk enrichment with dietary carotenoids in gulls: the roles of pigment acquisition and utilization. Funct. Ecol. **16**, 445-453. (10.1046/j.1365-2435.2002.00648.x)

[72] Lietz G, Lange J, Rimbach G. 2010 Molecular and dietary regulation of β, β-carotene 15, 15′-monooxygenase 1 (BCMO1). Arch. Biochem. Biophys. **502**, 8-16. (10.1016/j.abb.2010.06.032) 20599666

[73] Tompkins LM, Wallace AD. 2007 Mechanisms of cytochrome P450 induction. J. Biochem. Mol. Toxicol. **21**, 176-181. (10.1002/jbt.20180) 17936931

[74] Yang TB, Ooraikul BF. 2001 Studies on germination conditions and antioxidant contents of wheat grain. Int. J. Food Sci. Nutr. **52**, 319-330. (10.1080/09637480120057567) 11474896

[75] Smutny T, Mani S, Pavek P. 2013 Post-translational and post-transcriptional modifications of pregnane X receptor (PXR) in regulation of the cytochrome P450 superfamily. Curr. Drug Metab. **14**, 1059-1069. (10.2174/1389200214666131211153307) 24329114 PMC3914715

[76] Hill GE. 1995 Seasonal variation in circulating carotenoid pigments in the house finch. The Auk **112**, 1057-1061. (10.2307/4089042)

[77] Del Val E, Negro JJ, Garrido-Fernández J, Jarén M, Borras A, Cabrera J, Senar JC. 2014 Seasonal variation of red carotenoid pigments in plasma of wild crossbill males *Loxia curvirostra*. J. Ornithol. **155**, 211-218. (10.1007/s10336-013-1002-z)

[78] Del Val E, Senar JC, Garrido-Fernández J, Jarén M, Borràs A, Cabrera J, Negro JJ. 2009 The liver but not the skin is the site for conversion of a red carotenoid in a passerine bird. Naturwissenschaften **96**, 797-801. (10.1007/s00114-009-0534-9) 19357818

[79] Olson VA, Owens IPF. 2005 Interspecific variation in the use of carotenoid-based coloration in birds: diet, life history and phylogeny. J. Evol. Biol. **18**, 1534-1546. (10.1111/j.1420-9101.2005.00940.x) 16313466

[80] Witschi E. 1961 Sex and secondary sexual characteristics. In Biology and comparative physiology of birds (eds A.J. Marshal), pp. 115-168. New York, NY: Academic Press.

[81] Alonso-Alvarez C, Perez-Rodriguez L, Garcia JT, Vinuela J. 2009 Testosterone-mediated trade-offs in the old age: a new approach to the immunocompetence handicap and carotenoid-based sexual signalling. Proc. R. Soc. B **276**, 2093-2101. (10.1098/rspb.2008.1891) PMC2677252 19324780

[82] Martínez-Padilla J, Pérez-Rodríguez L, Mougeot F, Ludwig S, Redpath S. 2014 Intra-sexual competition alters the relationship between testosterone and ornament expression in a wild territorial bird. Horm. Behav. **65**, 435-444. (10.1016/j.yhbeh.2014.03.012) 24698833

[83] Bhujle BV, Nadkarni VB. 1976 Steroid dehydrogenases in the adrenal gland of four species of birds: a histochemical study. Histochem. J. **8**, 591-596. (10.1007/BF01003960) 993051

[84] Fevold H, Pfeiffer E, Rice J. 1973 Steroid biosynthesis by phalarope (*Steganopus tricolor* and *Lobipes lobatus*) adrenal tissue. Gen. Comp. Endocrinol. **21**, 353-357. (10.1016/0016-6480(73)90067-1) 4753363

[85] Freking F, Nazairians T, Schlinger BA. 2000 The expression of the sex steroid-synthesizing enzymes CYP11A1, 3β-HSD, CYP17, and CYP19 in gonads and adrenals of adult and developing zebra finches. Gen. Comp. Endocrinol. **119**, 140-151. (10.1006/gcen.2000.7503) 10936034

[86] Tanabe Y, Yano T, Nakamura T. 1983 Steroid hormone synthesis and secretion by testes, ovary, and adrenals of embryonic and postembryonic ducks. Gen. Comp. Endocrinol. **49**, 144-153. (10.1016/0016-6480(83)90018-7) 6826048

[87] Soma KK, Rendon NM, Boonstra R, Albers HE, Demas GE. 2015 DHEA effects on brain and behavior: insights from comparative studies of aggression. J. Steroid Biochem. Mol. Biol. **145**, 261-272. (10.1016/j.jsbmb.2014.05.011) 24928552

[88] Kero J, Poutanen M, Zhang F-P, Rahman N, McNicol AM, Nilson JH, Keri RA, Huhtaniemi IT. 2000 Elevated luteinizing hormone induces expression of its receptor and promotes steroidogenesis in the adrenal cortex. J. Clin. Invest. **105**, 633-641. (10.1172/JCI7716) 10712435 PMC289173

[89] Cantarero A, Pérez-Rodríguez L, Romero-Haro AÁ, Chastel O, Alonso-Alvarez C. 2019 Carotenoid-based coloration predicts both longevity and lifetime fecundity in male birds, but testosterone disrupts signal reliability. PLoS ONE **14**, e0221436. (10.1371/journal.pone.0221436) 31442265 PMC6707625

[90] Pike TW, Blount JD, Bjerkeng B, Lindström J, Metcalfe NB. 2007 Carotenoids, oxidative stress and female mating preference for longer lived males. Proc. R. Soc. B **274**, 1591-1596. (10.1098/rspb.2007.0317) PMC2169282 17439854

[91] Andersson S. 1989 Sexual selection and cues for female choice in leks of Jackson's widowbird *Euplectes jacksoni*. Behav. Ecol. Sociobiol. **25**, 403-410. (10.1007/BF00300186)

[92] Andersson S, Pryke SR, Örnborg J, Lawes MJ, Andersson M. 2002 Multiple receivers, multiple ornaments, and a trade-off between agonistic and epigamic signaling in a widowbird. Am. Nat. **160**, 683-691. (10.1086/342817) 18707516

[93] Hill GE. 2000 Energetic constraints on expression of carotenoid-based plumage coloration. J. Avian Biol. **31**, 559-566. (10.1034/j.1600-048X.2000.310415.x)

[94] Lindsey WR, Mendonça R, Slight MW, Prager M, Andersson MX, Mundy NI, Andersson S. 2022 Data from: Seasonal but not sex-biased gene expression of the carotenoid ketolase, *CYP2J19*, in the sexually dichromic southern red bishop (*Euplectes orix*). Dryad Digital Repository. (10.5061/dryad.ht76hdrjg) PMC9346373 35937912

[95] Lindsay WR, Mendonça R, Slight MW, Prager M, Andersson MX, Mundy NI, Andersson S. 2022 Seasonal but not sex-biased gene expression of the carotenoid ketolase, *CYP2J19*, in the sexually dichromic southern red bishop (*Euplectes orix*). Figshare. (10.6084/m9.figshare.c.6114863) PMC9346373 35937912

